# Tandem-repeat modules in phytopathogenic effectors driver their evolution and diversification

**DOI:** 10.1007/s44154-024-00208-3

**Published:** 2025-02-05

**Authors:** Hui Li

**Affiliations:** Key Laboratory of Seed Innovation, Institute of Genetics and Developmental Biology, Chinese Academy of Sciences, Beijing, 100101 China

**Keywords:** *Xanthomonas*, *Phytophthora*, Effectors, Tandem-repeat modules, TAL effectors, (L)WY effectors

## Abstract

Plant pathogens deliver effector proteins into both the host apoplast and host cells. These effectors function to colonize the host typically by altering host physiology or by subverting plant immune responses. The host plants have evolved intracellular nucleotide-binding site leucine-rich repeat (NBS-LRR) immunoreceptors that directly or indirectly recognize specific effector(s) to trigger plant immunity that prevents colonization. To circumvent effector-triggered immunity, adapted pathogens rely on constantly effectors evolution to further enhance susceptible host colonization. During the past few years, evidence has arisen that many effectors containing tandem repeat modules are particularly prone to rapid evolution through module insertion/deletion/shuffling, point mutations or adoption of other function domains. In this review, we highlight the diverse function of two modular effectors: TAL effectors in prokaryotic bacteria, (L) WY effectors in eukaryotic oomycetes, focus on new insights and the potential role of modularity in effector evolution, and discuss avenues for future research.

## Introduction

A number of specialized proteins, known as effectors, are released into the tissues of host plants by a wide range of plant-associated pathogens, including bacteria, fungi, oomycetes, nematodes and insects (Deslandes and Rivas 2012; Bozkurt et al. 2012). These effectors play key roles in facilitating colonization by altering physiological processes in the host plants or by manipulating their immune defenses (Hogenhout et al. 2009). In response, certain host plants have evolved complex immune systems with receptor proteins that are able to detect, either directly or indirectly, one or more of these effectors or the changes they cause in host targets (Cui et al. 2015; Böhm et al. 2014; Jones and Dangl 2006). This recognition triggers robust immune responses aimed at thwarting the pathogen's colonization efforts. To circumvent these immune barriers or to gain additional advantages in colonizing susceptible hosts, plant-associated pathogens often undergo effector modification through the process of adaptive evolution. This evolution is driven by the intense selective pressure of the host immune system, resulting in the appearance of novel, altered or enhanced effector functions that enhance the pathogen's colonizing capabilities (Dong et al. 2014; Win et al. 2007; Stergiopoulos et al. 2007; Deslandes and Rivas 2012; Bozkurt et al. 2012).

The effectors produced by plant-associated pathogens exhibit a diverse array of characteristics. Many of these proteins possess a signal peptide that directs their secretion or delivery into the host, ensuring their targeted deployment (Deslandes and Rivas 2012; Bozkurt et al. 2012). The tandem repeat module is a common feature of effector proteins produced by many pathogens (Franceschetti et al. 2017; Dong and Ma 2021). For example, effectors from the bacterial wilt pathogen, *Ralstonia solanacearum*, such as RipAP, RipBB, RipBC, and RipY, feature ankyrin repeats (Peeters et al. 2013). A number of effectors from *R. solanacearum* (RipG1-RipG7), and *Xanthomonas* species (XopAC, XopAE, and XopL) contain leucine-rich repeat (LRR) domains (Peeters et al. 2013; Xu et al. 2008; White et al. 2009). Effectors from both *R. solanacearum* and *Xanthomonas* species, including RipS1-RipS8, XopAD, and XopN, are characterized by the presence of HEAT/armadillo repeats (Peeters et al. 2013; White et al. 2009). In addition, the poplar leaf rust fungus *Melampsora larici-populina* produces three effectors known as Chloroplast-targeted protein 1–3 (CTP1-3). These proteins are characterized by carrying two or three amphipathic imperfect near-tandem repeats (Petre et al. 2016). A prominent example of modular effectors is the transcription activator-like (TAL) effector family produced by the bacterium *Xanthomonas* species. These effectors possess repeating modules, each containing variant residues that enable them to bind specifically to host DNA sequences of different lengths and nucleotide compositions (Timilsina et al. 2020; Perez-Quintero 2019). By rearranging these repeat units in different combinations, TAL effectors can target and manipulate specific genes in the host plant, underlining their remarkable versatility and adaptability (Timilsina et al. 2020; Perez-Quintero 2019). Another notable class of effectors that feature tandem repeats is the (L)WY effector family, which is highly abundant in oomycete species belonging to the *Phytophthora* and downy mildew lineages (Ma et al. 2018; Mesarich et al. 2015; Dong and Ma 2021). These modular proteins are distinguished by their RXLR motif, a characteristic sequence that facilitates their translocation into host cells after being released from the signal peptide (Dong and Ma 2021). The effector domains of (L)WY effectors with various tandem repeat (L)WY modules are designed to target diverse biological processes and subcellular compartments within the host plant, ultimately contributing to the infection strategy and success of the pathogen (Dong and Ma 2021; Li et al. 2023). Here, we focus on the TAL effectors in bacteria and (L)WY effectors in oomycete to discuss recent findings on the function and evolution of these two families of tandem repeat-containing effectors and to compare their commonalities amid differences.

## TAL effectors in bacteria

**Fig. 1:**
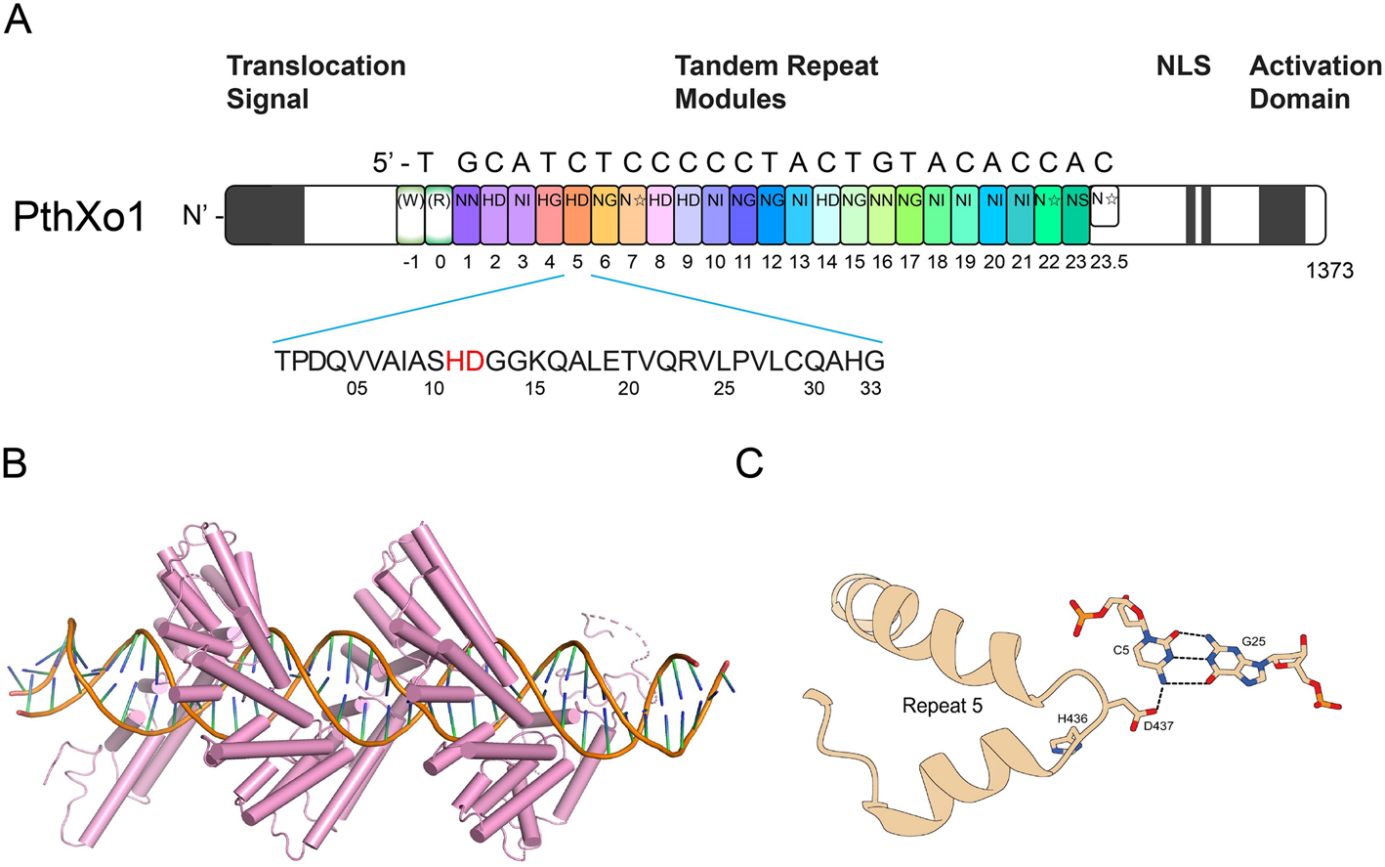
The domain organization and structure of PthXo1. **A** The domain organization of a TAL effector, PthXo1, contains N-terminal signals for bacterial type III secretion, tandem repeats specifying the target nucleotide sequence, nuclear localization signals (NLS) and a C-terminal region required for transcriptional activation. PthXo1 contains 23.5 canonical repeats. **B** The crystal structure of the PthXo1-dsDNA complex. The pink represents the PthXo1 protein. **C** Topology and contacts between repeat 5 of PthXo1 with a cytosine in the structure. HD (repeat 5) forms a steric and electrostatic contact with cytosine

TAL effectors containing remarkable tandem repeat modules from the genus *Xanthomonas* species are proteins that are injected into the nucleus, bind to the specific sequence of the host genes and modulate their expression, which can either benefit the bacterial colonization or trigger host defense (Kay et al. 2007; Römer et al. 2007). These effectors usually contain an N-terminal translocation signal region that is required for their secretion via the type III secretion system (T3SS), a central DNA-binding domain responsible for sequence specificity, a C-terminal putative motif resembling monopartite nuclear localization signal (NLS), and a C-terminal acidic activation domain (AD) responsible for gene modulation (Boch and Bonas 2010) (Fig. 1A).

In each TAL effector, the DNA-binding domain typically consists of a variable number of highly conserved tandem repeat modules of 33 to35 residues in length, which mediate DNA recognition. The nucleotide specificity of the repetitive module in TAL effector is encoded by two adjacent residues, positioned at the 12th and 13th amino acid positions, collectively known as the repeat variable diresidues (RVDs) (Boch et al. 2009; Boch and Bonas 2010). The recognition codes between RVDs and DNA bases have been established experimentally and computationally (Boch et al. 2009; Moscou and Bogdanove 2009). More than 20 types of RVDs have been identified in TAL effectors so far, but only seven types of repeats—His/Asp (HD), Asn/Gly (NG), Asn/Ile (NI), Asn/Asn (NN), Asn/Ser (NS), "N*" (representing a 33 residue repeat in which the RVD appears to lack its second residue), and Asn/Gly (HG)—together account for nearly 90% of all repeats and specifically encode for C, T, A, G/A, A/C/T/G, C/T, and T respectively (Boch et al. 2009; Boch and Bonas 2010). The crystal structure of the *Xanthomonas oryzae* TAL effector PthXo1 adopts a right-handed super helical structure with 11 repeat units per turn. Upon binding to DNA, it encircles the major groove, positioning the RVD loop on the inside of the helix in direct interaction with the DNA strands (Fig. 1B) (Mak et al. 2012). In the complexes of TAL proteins with double-stranded DNA, each TAL repeat is linked by two loops containing RVDs. The first RVD-containing loop stabilizes the protein backbone through hydrogen bonding, while the second loop establishes base-specific contacts with the dsDNA (Fig. 1C). These interactions enable TAL effectors to adopt a super helical conformation that tracks along the dsDNA (Mak et al. 2012). For now, several other crystal structures of TAL effectors have been solved, including PthA (Murakami et al. 2010), AvrBs3 (Stella et al. 2013), a TALE-like protein from *Paraburkholderia rhizoxinica* (Stella et al. 2014), which not only shed light on the mechanisms behind DNA-binding specificity, but also made TAL effectors one of artificially engineering tools (Deng et al. 2012b, 2012a; Yin et al. 2012; Gao et al. 2012).

TAL effectors bind the promoters of various host susceptibility (*S*) genes in a sequence-specific manner, making them an ideal probe for identifying physiological processes that control plant susceptibility to bacteria. Several TAL effectors-associated *S* genes have been identified (Yang et al. 2006; Antony et al. 2010). One of the best studied examples of TAL effectors and their corresponding *S* genes are the TAL effectors of *X. oryzae pv. oryzae* (*Xoo*) and the *SWEET* genes of rice. SWEET proteins act as sugar uniporters, which mediate the import and efflux of sugars into and out of animal and plant cells (Chen 2014). Several TAL effectors of *Xoo* are known to target one of the three *SWEET* genes in rice (Yu et al. 2011; Yang et al. 2006; Tran et al. 2018; Antony et al. 2010). More specifically, the expression of *SWEET11* is induced by the strains encoding the TAL effector PthXo1, *SWEET13* is induced by PthXo2 and *SWEET14* is induced by any one of the following TAL effectors at distinct site of the *SWEET14* promoter: AvrXa7, PthXo3, TalC and TalF (Doucouré et al. 2018; Zhou et al. 2015; Bing and White 2004; Yang et al. 2006; Yu et al. 2011; Tran et al. 2018; Antony et al. 2010; Streubel et al. 2013). The sequence-specific manner of TAL effectors offers the potential to edit the *SWEET* promoter using CRISPR-Cas9 and engineer broad-spectrum resistance in rice (Oliva et al. 2019). TAL effectors also can target crucial host transcription factors, including the ethylene response factor OsERF123 and the leucine zipper domain (bZIP) transcription factor OsTFX1 in rice (Wang et al. 2017; Sugio et al. 2007; Tran et al. 2018), the basic helix-loop-helix (bHLH) UPA20 in pepper (Kay et al. 2007), and the LOB (lateral organ boundary) family of the transcription factors in Citrus (Zhang et al. 2017; Hu et al. 2014; Doucouré et al. 2018), two bHLH transcription factors in tomato (Schwartz et al. 2017).

As expected in an arms race, plants develop different strategies to effectively reduce bacterial virulence. A simpler strategy, referred to as "loss-of-susceptibility," involves mutating the TAL effector binding element (EBE) of the *S* gene to prevent it binding of TAL effector. This resistance mechanism underlies some of the recessive resistant (*R*) genes already described, including *xa13* (Yang et al. 2006; Chu et al. 2006), *xa25* (Zhou et al. 2015) and *xa41* (Hutin et al. 2015b). These mutations either occur within the EBE of the *SWEET* genes and block the binding of the TAL effector (Hutin et al. 2015a). In addition, TALE-mediated ETI has been used as another counter-attack strategy for plants with *NBS-LRR* resistance genes identified in tomato and rice. XA1 (Ji et al. 2016; Yoshimura et al. 1998) and Bs4 (Schornack et al. 2004), which are NBS-LRR resistance proteins from rice and tomato, recognize multiple TALEs.

## (L)WY effectors in oomycete

**Fig. 2:**
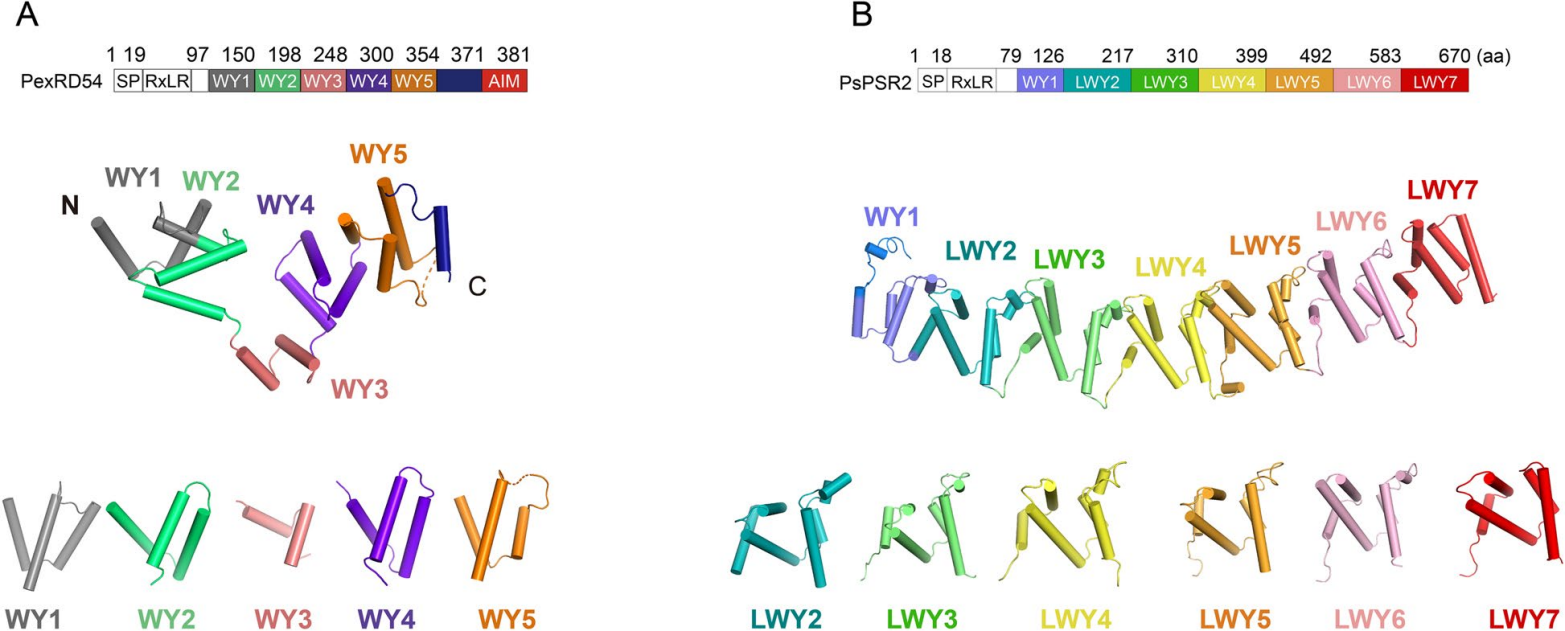
The crystal structure of two (L)WY effectors, PexRD54 (left) and PsPSR2 (right). **A** The crystal structure of WY effector, PexRD54, which contains five WY tandem repeat modules. Top: Schematic representation of the domain organization of PexRD54. Middle: The crystal structure of PexRD54. The individual WY module is displayed in a specific color. Bottom: The structure of individual WY module. **B** The crystal structure of WY effector, PsPSR2, which contains seven (L)WY tandem repeat modules. Top: Schematic representation of the domain organization of PsPSR2. Middle: The crystal structure of PsPSR2. The individual (L)WY module is displayed in a specific color. Bottom: The structure of individual LWY module. SP, secretion signal peptide; RXLR, a translocation motif of *Phytophthora* effectors

The well-known class of oomycete effectors containing tandem repeat modules are the RXLR effectors, which are strongly enriched in *Phytophthora *and downy mildew lineages. The family of modular effectors contains an N-terminal RXLR motif consisting of arginine (R), any amino acid (X), leucine (L) and arginine (R), which is critical for the translocation of these effectors (Whisson et al. 2007; Dou et al. 2008). The RXLR motif is followed by the C-terminal effector domain, which shares a set of structurally conserved but highly degenerate folds, termed the WY modules (Jiang et al. 2008). Protein structural analysis subsequently revealed that two hydrophobic residues (Trp (W) and Tyr (Y)) are buried within the core of the helical bundle in each WY module. The WY modules appear to be prevalent in the *Peronosporales* and were predicted by bioinformatic methods to be present in approximately 44% of *Phytophthora infestans* RXLR effectors and a quarter of *Hyaloperonospora arabidopsidis* RXLR effectors. (Goss et al. 2013; Franceschetti et al. 2017; Boutemy et al. 2011; Win et al. 2012). Effectors containing the WY module exist in a variety of forms including single, duplicated, dimeric or tandem repeats (Goss et al. 2013; Franceschetti et al. 2017; Boutemy et al. 2011; Win et al. 2012). Structural analysis of the *P. infestans* effector PexRD54 shows that it contains five tandem repeat WY modules (Fig. 2A), whereas the *P. sojae* effector PsAvh240 consists of two WY modules (Dagdas et al. 2016).

Recently, based on the structure of another WY effector *Phytophthora* suppressor of RNA silencing 2 (PsPSR2), a new LWY module was defined (Fig. 2B) (He et al. 2019). PsPSR2 is composed of seven tandem repeat modules including an N-terminal WY (WY1) and six LWY modules (LWY2-LWY7). Different from the WY module, each LWY module consists of a conserved five-helix fold with two hydrophobic pockets and the newly defined L motif, a region of about 45 amino acids, contains several conserved residues (often leucine) which are responsible for forming the additional hydrophobic core and interacts with an internal loop from the preceding module (He et al. 2019). This 'joint-like' connection provides directional links that stabilize the concatenation of individual modules, resulting in the highly organized, rod-shaped structure of PsPSR2 (Fig. 2B) (He et al. 2019). The effectors with only WY modules lack conservation in the internal loop due to the absence of the L motif, resulting in different linkage mechanisms between adjacent WY module pairs, such as in the case of PexRD54 (Fig. 2A). As a result, the effectors with only WY modules may exhibit variable protein shapes. Meanwhile, the effectors with LWY modules are generally longer in length than effectors without the (L)WY tandem repeat modules or with only WY modules, resulting in larger surface areas that promote the ability to interact with proteins or other molecules in the host. Approximately 15% of the total number of RXLR effectors consists of the LWY modules in five *Phytophthora* species, suggesting that this module is also prevalent in *Phytophthora* effectors (He et al. 2019). (L)WY modules share a low degree of amino acid sequence conservation, limited to a small number of buried residues that are essential for maintaining the fold of individual modules and the overall structure. Functional differentiation of (L)WY effectors may be facilitated by the high degree of sequence plasticity of surface residues and different chimeras of (L)WY modules. Indeed, effectors with different WY/LWY modules or combinations of modules have different molecular functions in the host plant.

A *P. sojae* effector PsAvh240 contains two WY modules in the C-terminal region (Guo et al. 2019). The first WY module is responsible for its plasma membrane localization and interaction with the soybean aspartic protease 1 (GmAP1), whereas the second WY module is responsible for homodimerization via a molecular handshake arrangement and the effector's repression of GmAP1 secretion to promote infection (Guo et al. 2019). The first two WY/LWY repeat modules in PsPSR2 are sufficient to mediate interaction with a host target protein, Double-Stranded RNA Binding Protein 4 (DRB4), which suppresses trans-kingdom RNAi to promote disease susceptibility (Hou et al. 2019). Recently, the function of other LWY modules of PsPSR2 has been demonstrated, indicating that the LWY2-LWY3 combination of PsPSR2 forms a functional module to recruit the serine/threonine protein phosphatase 2A (PP2A) core enzyme in plant hosts, and that the C-terminal LWY modules may be responsible for the recruitment of PSR2-PP2A complex substrates (Li et al. 2023). Interestingly, while a cluster of LWY effectors sharing PP2A-interacting LWY modules at the amino terminus all have the ability to hijack the host PP2A core enzyme and form functional holoenzymes, they possess C-terminus with a divergent combination of LWY modules and may recruit distinct sets of phosphoproteins to the effector-PP2A complexes in the host (Li et al. 2023). *Hyaloperonospora parasitica* effector ATR1, which contains two LWY modules, not only confers enhanced virulence to *Pst* DC3000 on susceptible Arabidopsis accessions, but could also be recognized by an Arabidopsis NLR Recognition of *Peronospora parasitica* 1 (RPP1) that carries Toll-like interleukin-1 (TIR) receptor domains and elicits a plant immune response (Sohn et al. 2007; Ma et al. 2020; Chou et al. 2011). The linear arrangement of the two tandem repeat LWY modules in ATR1, which folds it into an extended, non-globular overall structure, and the lack of significant sequence identity between the two repeats allow it to evade recognition by rapid evolution and adopt diverse virulence functions (Krasileva et al. 2010; Ma et al. 2020). The broadly conserved WY module-containing effectors in several *Phytophthora* species, AVRamr3 and its homologues, share similar structures despite extensive sequence variation, but all could be recognized by a potato late blight resistance protein Rpi-amr3, demonstrating that the common fold of the WY module together with sequence polymorphisms on the effector surface may determine the interaction with host proteins (Lin et al. 2022).

In addition, the WY module may employ functional domains to associate with host proteins to promote disease. The crystal structure of the *P. infestans* effector protein PexRD54 revealed that it contains five WY domains and a short linear motif known as the ATG8-interacting motif (AIM), which binds to the host autophagy protein ATG8CL to stimulate autophagosome formation and subverts host vesicle trafficking (Dagdas et al. 2016; Pandey et al. 2021). The structural arrangement of PexRD54 demonstrates that a tandem (L)WY module or multiple (L)WY combinations could serve as a scaffold to provide a functional domain for interaction with host proteins.

## Tandem repeat modules may contribute to effectors diversity

**Fig. 3:**
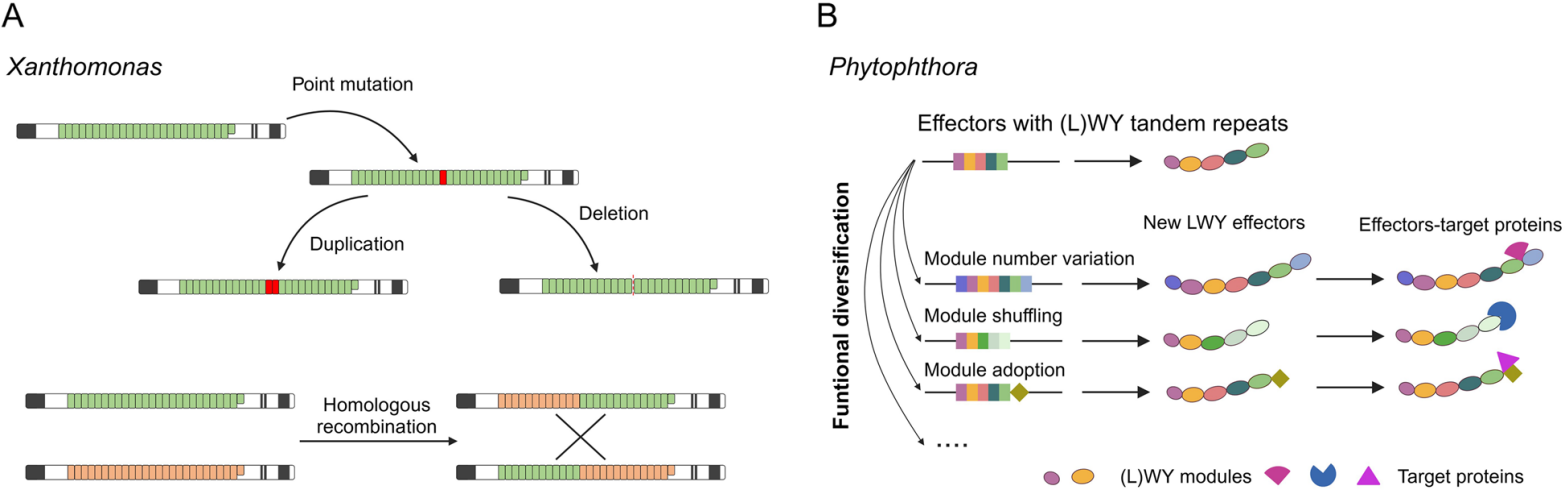
The evolutionary mechanisms of TAL effectors and LWY effectors. **A** Mechanisms described to generate repeated sequence variation in TAL effectors. **B** Tandem repeat modules -driven effector evolution through the arrangement of the WY/LWY modules. This modular architecture facilitates extensive polymorphism, arising from the insertion, deletion, and shuffling of modules, as well as the integration of additional functional domains

Tandem repeat modules can evolve in a number of ways, including by changes in the number or order of repeat modules (duplication, and insertions/deletions), and by amino acid substitutions, particularly of residues on the surface of the module (Fig. 3A) (He et al. 2019; Perez-Quintero 2019; Dong and Ma 2021). In addition, recombination appears to be particularly common, with multiple repeats set often swapped.

Indeed, there has since been evidence for intra- and inter- genic recombination events among TAL effectors (Yang et al. 2005; Yang and Gabriel 1995). The first example of how repeat module variability could confer an adaptive advantage on effectors was identified through the experimental manipulation of AvrBs3, a TAL effector from *Xanthomonas euvesicatoria* (Herbers et al. 1992). AvrBs3 binds to the promoter of *UPA20*, a host gene encoding a basic helix-loop-helix transcription factor, to induce hypertrophy of the plant cell in a compatible interaction with pepper plants (Moscou and Bogdanove 2009; Kay et al. 2007). Whereas AvrBs3 binds to the promoter of *Bs3*, a pepper gene that encodes an executor resistance protein to induce host immunity in an incompatible interaction with pepper plants (Römer et al. 2009, 2007). To explore whether the repeat module in AvrBs3 provide a source of functional diversity, many AvrBs3 deletion derivatives that differed in the number of their repeat modules were generated. And most of the deletion derivatives lost their ability to induce Bs3-dependent immunity, however, others could gain a new host specificity and induce immunity in the pepper plants with Bs3-E, an allele of Bs3 (Herbers et al. 1992). In addition, the repeat module swaps between AvrBs3 and AvrXa7 provide that the potential for a virulence effector to lose avirulence activity but retain effector virulence function, also suggesting that repeat module variability could confer TAL effectors with an adaptive advantage and rapid evolution (Yang et al. 2005). The recombination of repeat modules in/between TAL effectors could generate novel effectors that target different host genes by altering the DNA binding specificity (Yang et al. 2005; Yang and Gabriel 1995).

For (L)WY effectors, sequence diversification has been shown to play a particularly important role in driving the evolution of tandem repat modules, with sequence conservation of (L)WY modules restricted to a small number of buried residues (He et al. 2019; Jiang et al. 2008; Li et al. 2023). This feature provides a conservative structural framework for these effectors, enabling their divergent evolution and facilitating functional differentiation (Fig. 3B) (He et al. 2019; Jiang et al. 2008; Li et al. 2023). In addition, many (L)WY effectors are a combination of different (L)WY modules, adding another layer of potential to develop novel activities (He et al. 2019; Jiang et al. 2008; Li et al. 2023). The cluster of PP2A-interacting effectors elegantly demonstrated how (L)WY effectors combine the common module with different LWY modules, leading to the diversity in an effector repertoire. Moreover, (L)WY modules also combined with other functional motif(s), further expanding the diversity of effector function (Pandey et al. 2021; Dagdas et al. 2016).

## Future perspectives — Precision engineering to create resistant crops

Despite the discovery of two families of tandem repeat-containing effectors: TAL effectors in prokaryotic bacteria, (L) WY effectors in eukaryotic oomycetes, and that many of these effector proteins enhance virulence, we are still in the early stages of understanding how these repetitive modules contribute to virulence. From the limited number of examples analyzed, it is becoming apparent that such repeats provide a mechanism for adaptation through changes in repeat order or number by intra- and inter-genic recombination, and slippage during replication, leading to insertions or deletions (Perez-Quintero 2019; Dong and Ma 2021). However, the current challenge is to develop assays that are sensitive sufficient to detect subtle differences in the function of tandem repeat-containing effectors that are caused by variations in individual repeats, repeats combination, or specific repeat arrangements. Understanding how these variations confer a selective advantage to the pathogen in its co-evolutionary arms race with the host is crucial for developing effective strategies to combat plant diseases caused by pathogens. This requires a deep understanding of the molecular mechanisms underlying effector function and how they evolve over time.

Currently, our understanding of the structural features and molecular mechanisms of TAL effectors and (L)WY effectors empower scientists the potential to precisely manipulate these effector proteins through techniques such as gene editing, protein direct evolution, de novo design, and artificial intelligence (AI). Protein direct evolution and de novo protein design methodologies enable the creation of proteins featuring novel folds not previously observed in nature. Recent advancements have led to the successful development of various tandem repeat proteins using protein direct evolution or de novo design techniques (Harris et al. 2013; Doyle et al. 2023; Jiang et al. 2024). Notably, TAL and (L)WY effectors, which are characterized by their specific repeat numbers, DNA binding sites, interacting proteins, and functional attributes, exhibit significant potential for being crafted through protein direct evolution, de novo design integrated with artificial intelligence. With the advancement of AI tools, such as AlphaFold, large-scale structure prediction and functional characterization of tandem repeat-containing effectors have become feasible. These AI tools have enabled a deeper understanding of the precise roles that repeat modules play in the function and adaptive evolution of these effectors. Recently, a significant number of short TALE-like repeat (STAR) DNA-binding proteins have been identified and functionally characterized, with AlphaFold2/3 playing a crucial role in this process (Hu et al. 2024). It is anticipated that as AI tools continue to evolve, they will play an increasingly pivotal role in future studies of effectors containing tandem repeat modules, which form conserved folds with varying levels of sequence similarity, such as the higher similarity observed in TAL effectors and the lower similarity in WY effectors. These multidisciplinary approaches not only enhance our ability to create disease-resistant crops but also accelerate the pace of agricultural innovation, ensuring global food security in the face of ever-evolving pathogen threats.

## Data Availability

All data generated or analyzed during this study are included in this published article.

## Abbreviations

AD: Activation domain
AI: Artificial intelligence
AIM: ATG8-interacting motif
bHLH: Basic helix-loop-helix
DRB4: Double-Stranded RNA Binding Protein 4
EBE: Effector binding element
GmAP1: Soybean aspartic protease 1
L: Leucine
NBS-LRR: Nucleotide-binding site leucine-rich repeat
NLS: Nuclear localization signal
PP2A: Protein phosphatase 2A
PsPSR2: Phytophthora Suppressor of RNA silencing 2
R: Arginine
R genes: Resistant genes
RPP1: Recognition of Peronospora parasitica 1
RVDs: Repeat variable diresidues
S genes: Susceptibility genes
STAR: Short TALE-like repeat
TAL: Transcription Activator-like
TIR: Toll-like interleukin-1
T3SS: Type III secretion system
W: Trp
X: Amino acid
Xoo: Xanthomonas. oryzae pv. oryzae
Y: Tyr
